# Extensive Degradation and Low Bioavailability of Orally Consumed Corn miRNAs in Mice

**DOI:** 10.3390/nu10020215

**Published:** 2018-02-15

**Authors:** Haiqiu Huang, Cindy D. Davis, Thomas T. Y. Wang

**Affiliations:** 1 Diet, Genomics and Immunology Laboratory, Beltsville Human Nutrition Research Center, USDA-ARS, Beltsville, MD 20705, USA; tennisqiu@gmail.com; 2 Office of Dietary Supplements, National Institutes of Health, Bethesda, MD 20892, USA; davisci@mail.nih.gov

**Keywords:** corn, cross-kingdom transfer, C57BL/6 mouse, miRNA, periodate oxidation

## Abstract

The current study seeks to resolve the discrepancy in the literature regarding the cross-kingdom transfer of plant microRNAs (miRNAs) into mammals using an improved miRNA processing and detection method. Two studies utilizing C57BL/6 mice were performed. In the first study, mice were fed an AIN-93M diet and gavaged with water, random deoxynucleotide triphosphates (dNTP) or isolated corn miRNAs for two weeks (*n* = 10 per group). In the second study, mice were fed an AIN-93M diet, or the diet supplemented with 3% fresh or autoclaved corn powder for two weeks (*n* = 10 per group). Corn miRNA levels were analyzed in blood and tissue samples by real-time PCR (RT-PCR) following periodate oxidation and β elimination treatments to eliminate artifacts. After removing false positive detections, there were no differences in corn miRNA levels between control and treated groups in cecal, fecal, liver and blood samples. Using an in vitro digestion system, corn miRNAs in AIN-93M diet or in the extracts were found to be extensively degraded. Less than 1% was recovered in the gastrointestinal tract after oral and gastric phases. In conclusion, no evidence of increased levels of corn miRNAs in whole blood or tissues after supplementation of corn miRNAs in the diet was observed in a mouse model.

## 1. Introduction

MicroRNA (miRNA) are a group of small, functional, non-protein coding RNA oligonucleotides that were discovered two decades ago and are universally found in microorganisms, plants, and animals [1,2]. The miRNAs have been shown to mediate 30% of the post-transcriptional silencing in mammals and modulate a wide range of critical biological processes, including neuronal development, cell differentiation, apoptosis, proliferation, and immune response [1,2,3,4,5]. In 2012, Zhang and colleagues reported a novel cross-kingdom uptake of intact rice miRNAs (miR156a and miR168a) via dietary consumption into circulation and organs of humans and mice [6]. More importantly, the rice miR168a was reported to directly down-regulate the expression of a cholesterol regulation-related gene LDLRAP1 in the liver [6]. This result suggests ingestion of plant miRNA may influence physiology and health in humans. The importance of this finding was signified by the number and breadth of follow-up studies [7,8,9,10,11,12,13,14,15,16,17,18]. However, various follow up bioinformatic studies, in vitro and in vivo studies to identify the cross-kingdom transfer of plant miRNAs and the presence of exogenous miRNAs in human or mammalian circulation or organs lead to mixed results [7,8,9,10,11,12,13,14,15,16,17,18]. At present, no definitive conclusion has been reached regarding the extent and prevalence of dietary plant miRNAs entering circulation. 

The detection of plant miRNAs in human or animal samples can be challenging and confounded by the potential artifacts introduced in the sample preparation, sequence detection, and difficulties in delineating the origin of miRNAs [15,16]. These inherent problems related to plant miRNA analysis may contribute in part to the difficulty in validating/confirming cross-kingdom regulation by plant miRNAs. A good marker for the plant origin of miRNAs for specific detection of plant miRNAs in biological samples is the distinct methylation patterns on the 3′ ends of plant and mammalian miRNAs [19]. Taking advantage of 3′ protective property of plant miRNA, we previously reported that periodate oxidation and β elimination reaction treatment specifically targets the unmethylated 3′ end of miRNAs to eliminate the artifacts in PCR detection of plant miRNAs in biological samples [20]. Hence, we believe that analysis using this method may help us validate the presence of plant miRNA in biological samples and elucidate bioavailability of plant miRNA in vivo. 

The present study seeks to test the hypothesis that dietary plant-derived miRNA can survive the gastrointestinal (GI) tract and be taken up into circulation and tissues in vivo. This study utilized corn as the source of dietary plant miRNAs. Common plant miRNAs, such as miR156a, miR164a, and miR167a, are detected at high levels in corn [21]. More importantly, corn and its processed products are the most widely used/consumed ingredients in foods [22,23], therefore the cross-kingdom transfer of these miRNAs could bear significant biological, health and economic impacts. Corn or corn miRNA extract were incorporated into rodent diet. An autoclaving-base method was developed to degrade corn miRNAs to be use as a control treatment to eliminate the matrix effect as a variable. Corn miRNAs in the gastrointestinal tract, liver, and blood from animals fed a corn supplemented diet or gavaged with miRNA extracts from corn were determined using the periodate oxidation/β-elimination method. Our results indicate that corn miRNA was extensively degraded in the GI tract and that the uptake into circulation and tissues was minimal. 

## 2. Materials and Methods

### 2.1. Reagents and Chemicals

Tris hydrochloride (pH 9.0), sodium dodecyl sulfate (SDS), lithium chloride (LiCl), ethylenediaminetetraacetic acid (EDTA), phenol (pH 8.0), chloroform-isoamyl alcohol (24:1; *v*/*v*), phenol-chloroform-isoamyl alcohol (25:24:1; *v*/*v*/*v*), sodium acetate (3 M, pH 5.2), sodium chloride (NaCl), polyethylene glycol 8000, and pepsin were obtained from Sigma-Aldrich (St. Louis, MO, USA). RNA oligos with 3′ end 2′-*O*-methylation were synthesized by Integrated DNA Technologies, Inc. (Coralville, IA, USA).

Corn small RNA isolation. Fresh corn used in this study was purchased from a local market (Beltsville, MD, USA). Corn small RNAs were isolated according to a previously published protocol [24]. Briefly, 0.1 g of pulverized plant sample was added in a 1.5 mL microcentrifuge tube with 500 μL of LiCl extraction buffer and 500 μL of phenol pH 8.0. Extraction mixture was vortexed for 1 min and incubated for 5 min at 60 °C. Then the mixture was centrifuged at 20,000× *g* at 4 °C for 10 min. The upper phase was transferred to a new microcentrifuge tube and 600 μL of chloroform-isoamyl alcohol (24:1; *v*/*v*) was added. The mixture was vortexed and centrifuged, and the upper phase was transferred to a new microcentrifuge tube and incubated at 65 °C for 15 min. Then, 50 μL of 5 M NaCl and 63 μL of 40% polyethylene glycol 8000 (*w*/*v*) was added followed by incubation on ice for at least 30 min. The low molecular weight RNA was separated from the pellet which consisted of high molecular weight RNA and DNA by centrifugation. The supernatant was mixed with 500 μL of phenol-chloroform-isoamyl alcohol (25:24:1; *v*/*v*/*v*). The mixture was centrifuged, and the supernatant was transferred to a new microcentrifuge tube with 50 μL of 3 M sodium acetate pH 5.2 and 1200 μL of absolute ethanol. RNA samples were incubated at −20 °C overnight. The small RNA was precipitated, washed twice, and dried. Isolated RNA was resuspended in RNase-free water and kept in −80 °C. RNA concentration and purity were determined using Nanodrop 8000 Spectrophotometer (Thermo Fisher Scientific, St. Louis, MO, USA).

### 2.2. Animals, Diets, and Study Design

Male C57BL/6 mice (5-week old, Charles River, Wilmington, MA, USA) were acclimated for 1 week and given free access to water and AIN-93M diet (D10012M, Research Diet, Inc., New Brunswick, NJ, USA). Then the animals were randomly assigned to two subsequent studies (30 animals each). For study 1, all animals were fed a control diet (AIN-93M, 10% calorie from fat). The animals were divided into three treatments (*n* = 10/treatment) (1) control group (water); (2) random nucleotides (25 μg in 100 µL distilled water) (dNTP group); (3) purified small RNA isolated from corn kernel (25 μg in 100 µL distilled water) (Corn sRNA group). Random nucleotides and corn small RNA were administered by gavage using water as a vehicle and the control group was given 100 µL water. For study 2, the animals were assigned to the following three groups: (1) control diet (AIN-93M, 10% calorie from fat) (Control group); (2) AIN-93M + 3% autoclaved corn kernel powder (Autoclaved corn group); (3) AIN-93M + 3% fresh corn kernel powder (Fresh corn group) (Supplementary Materials Table S1). Corn kernel was incorporated into diets as a ground powder. The amounts of corn small RNA or corn kernel were calculated based on 1 serving/day of 166 g corn kernel for human consumption (National Nutrient Database for Standard Reference, Release 28, Agricultural Research Service-USDA) and a 5 g per day of food intake for the mouse. The powder was blended into the formulated diet by Research Diets, Inc. (New Brunswick, NJ, USA). Animals were single-housed in ventilated racks for the duration of the experiment. Fecal samples were collected daily. At the end of the two-week feeding period, blood was collected by cardiac puncture with syringes previously rinsed with potassium EDTA solution (15% *w*/*v*) and kept on ice. Contents of cecum and colon, and liver samples were collected, immediately frozen in liquid nitrogen. All samples were kept at −80 °C before analysis. This study was carried out in strict accordance with the recommendations in the Guide for the Care and Use of Laboratory Animals of the National Institutes of Health. The protocol was approved by the U.S. Department of Agriculture (USDA) Agricultural Research Service (ARS) Beltsville Area Institutional Animal Care and Use Committee (IACUC) (Protocol # 16-017). 

### 2.3. Plant miRNA Isolation and Detection

MiRNA isolation from the blood and tissue samples was performed using the mirVana™ miRNA Isolation Kit (with phenol) from Thermo Fisher Scientific (St. Louis, MO, USA) according to the manufacturer’s protocol. Plant miRNA was detected and quantitated using quantitative real-time PCR (qRT-PCR) as previously described [20]. Specific plant miRNA primers were purchased from Thermo Fisher Scientific (St. Louis, MO, USA) (TaqMan MicroRNA Assay: 000333, 000344, 000348, 241641_mat) and used for microRNA reverse transcription and detection. TaqMan MicroRNA Reverse Transcription Kit (Thermo Fisher Scientific, St. Louis, MO, USA) and the small RNA-specific RT primer from the TaqMan MicroRNA Assays were used to reverse transcribe complementary DNA. Five µL of 2 ng/µL RNA was used in reverse transcription and 1 µL of reverse transcription product was used in quantitative PCR. PCR was performed on ViiA7 Real-Time PCR System (Applied Biosystems, Foster City, CA, USA) using TaqMan Universal PCR Master Mix (Cat No.: 4304437) and miRNA-specific TaqMan primer from the TaqMan MicroRNA Assays. The following amplification parameters were used for PCR: 50 °C for 2 min, 95 °C for 10 min, and 40 cycles of amplification at 95 °C for 15 s and 60 °C for 1 min.

### 2.4. In Vitro Digestion of Plant miRNAs

An in vitro digestion model was used to mimic oral, gastric, and intestinal digestion of plant miRNAs and the procedure was adapted from a previously published protocol [25]. Rodent fecal samples were collected at the moment of excretion and immediately frozen in liquid nitrogen. Fecal pellets were re-suspended in LB broth in an anaerobic condition and vortexed to homogenize and release the fecal bacteria into the supernatant. The fecal suspension was then centrifuged at 800× *g* for 3 min to separate the fecal debris. Fecal bacteria were expanded in LB broth in anaerobic condition at 37 °C and cryopreserved in 25% glycerol in −80 °C before treatment. Briefly, the oral digestion phase: an electrolyte buffer (composed of KCl, KH_2_PO_4_, NaHCO_3_, NaCl, MgCl_2_(H_2_O)_6_, (NH_4_)_2_CO_3_, CaCl_2_(H_2_O)_2_) and 75 U/mL α-amylase at pH 7; the gastric digestion phase: an electrolyte buffer and 2000 U/mL pepsin at pH 3; the intestinal digestion phase: an electrolyte buffer, 100 U/mL pancreatin, and 10 mM bile at pH 7; the colonic phase: fecal bacteria culture in anaerobic condition (seeded at 1 × 10^7^ per mL) at 37 °C. Oral phase: 1 g/mL of AIN-93M diet with fresh corn, 25 µg/mL of corn miRNA extract, or 25 µg/mL of methylated or unmethylated miR171j were mixed with the oral phase digestion buffer at 1:1 (*v*/*v*) and incubated at 37 °C for 2 min. Gastric Phase: the oral mix, after incubation, was mixed with the gastric phase digestion buffer at 1:1 (*v*/*v*) and incubated at 37 °C for 2 h. Intestinal phase: the gastric mix, after incubation, was then mixed with the intestinal phase digestion buffer at 1:1 (*v*/*v*) and incubated at 37 °C for 2 h. Colonic phase: the intestinal mix, after incubation, was mixed with the colonic phase digestion fluid at 1:1 (*v*/*v*) and incubated at 37 °C in anaerobic condition overnight. Digestion samples were collected at each phase and flash frozen in liquid nitrogen and stored at −80 °C until analysis. MiRNAs were isolated using the mirVana™ miRNA Isolation Kit (with phenol) and plant miRNAs were detected using RT-PCR described above. Recovery of miRNAs was calculated as the percentage of miRNAs detected after each digestion phase to the number of miRNAs added at the beginning of the assay.

### 2.5. Statistical Analysis

All experiments were conducted in triplicate and representative data was reported as a mean ± standard deviation. A 0.005 amol to 500 fmol range of synthetic miRNAs were used to construct a standard curve in qRT-PCR. Linear regression and statistical analysis were performed using GraphPad Prism 6 (2015, GraphPad Software, La Jolla, CA, USA). Significance differences between means from treated groups compared to controls were determined using one-way analysis of variance (ANOVA) and Tukey’s Honestly Significant Difference (HSD) test. Statistical significance was defined at *p* ≤ 0.05.

## 3. Results

### 3.1. Food Intake, Body Weight and Levels of Corn miRNAs Consumption

**Figure 1:**
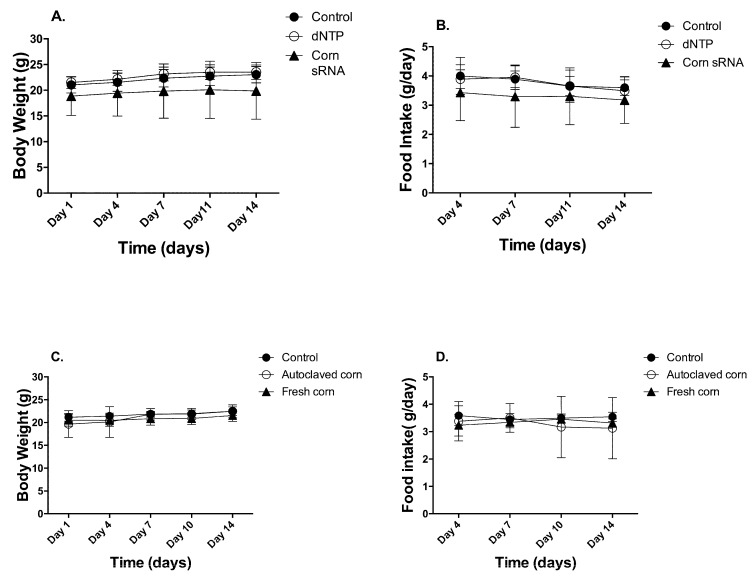
Animal body weight and food intake. Animal body weight and food intake were recorded twice a week and data were presented as Mean ± Standard Deviation (*n* = 10, (**A**,**B**) for 1st study and (**C**,**D**) for 2nd study).

Two animal studies were conducted to test our hypothesis. In study 1, animals received daily gavage of (1) 100 µL ultrapure water, (2) 100 µL of 250 ng/µL of random dNTP, and (3) 100 µL of 250 ng/µL of corn sRNA isolates. The isolates were pre-determined to contain 56.03 pg of miR156a, 13.2 pg of miR164a, and 1215.63 pg of miR167a per 25 µg of corn sRNA isolates. In study 2, animals were fed (1) AIN93M base diet, (2) AIN93M + 3% fresh corn powder, (3) AIN93M + 3% autoclaved corn powder. The diet supplemented with the fresh corn powder was determined to contain 173 pg of miR156a, 65 pg of miR164a, and 515 pg of miR167a per gram diet. No significant reduction of corn miRNA in the diet was detected between the start and end of the study (Supplementary Materials, Figure S1). Autoclaving corn powder at 121 °C for 30 min degraded 99%, 98%, and 97% of miR156a, miR164a, and miR167a, respectively (Supplementary Materials, Figure S2). The autoclaved corn powder was used as matrix control. For the corn sRNA extract study, no difference was observed in body weight and food intake throughout the two-week feeding period (Figure 1A,B). For corn powder supplementation study, no difference was observed in final body weight and food intake. Interestingly, total body weight gain over the 2-week feeding period was significantly lower in the fresh-corn fed animals (1.03 ± 0.89 g), comparing to that of autoclaved-corn fed animals (2.03 ± 0.68 g) (*p* < 0.05) (Figure 1C,D).

### 3.2. Analysis of Corn miRNAs in Blood and Liver

**Figure 2:**
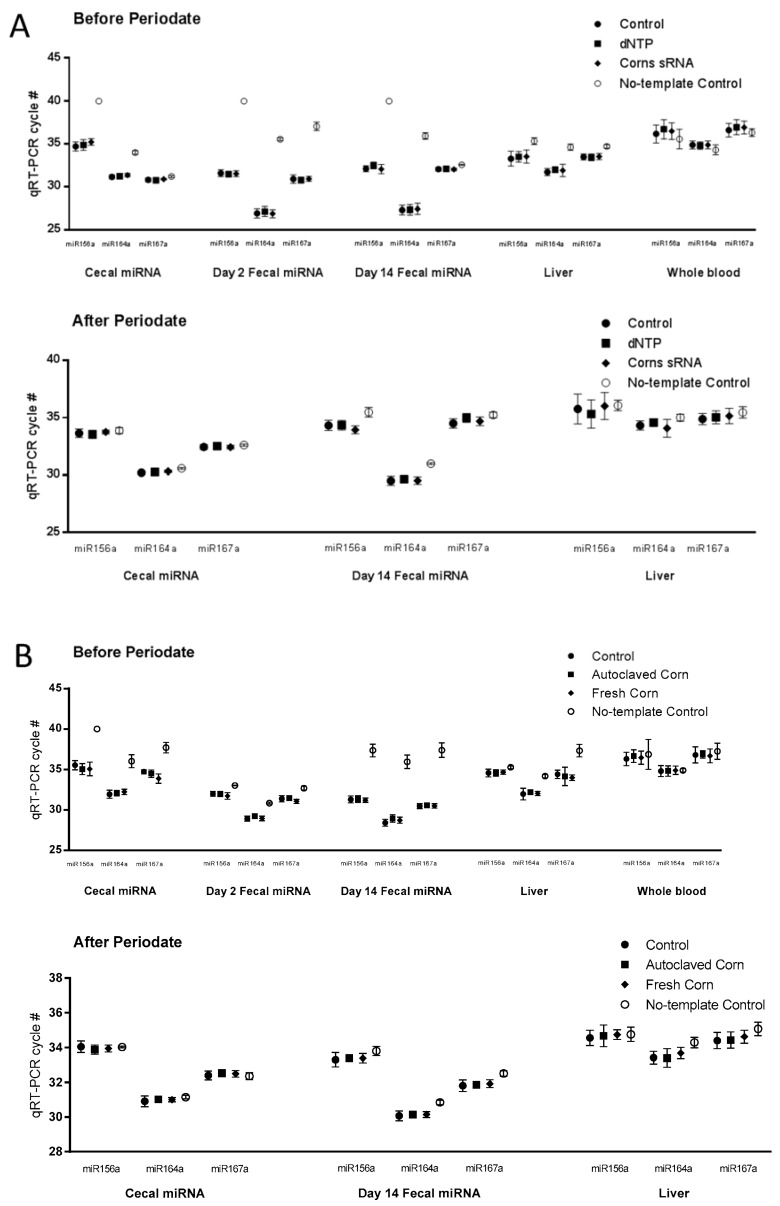
Plant miRNA detection in tissue samples. Plant microRNA (miRNA) were detected and quantified using qRT-PCR as described in Materials and Methods. Cycle numbers of real-time PCR (RT-PCR) before and after periodate oxidation treatment followed by β elimination were presented as Mean ± Standard Deviation (*n* = 10, (**A**) for 1st study and (**B**) for 2nd study).

Liver and whole blood samples from both studies were analyzed for plant miRNAs (miR156a, miR164a, and miR167a) as determined by the Ct values for specific miRNA from RT-PCR where higher Ct values indicate lower expression values. In study 1, using corn small RNA extracts, we found no differences in miRNA Ct values between the control, dNTP, and corn miRNA groups in the liver and whole blood samples (Figure 2A, upper panel). In the whole blood samples, the Ct values of the three experimental groups was the same as no-template control (Figure 2A, upper panel). By contrast, the Ct values of the liver samples were significantly lower than the no-template controls (Figure 2A upper panel). However, after periodate oxidation followed by β elimination, no differences were detected between the no-template control group and the experimental groups in the liver and blood samples (Figure 2A lower panel, Table 1). 

Similar observations were made in the liver and whole blood samples from the 2nd study using the fresh corn powder. No differences were detected in miRNA Ct values among the control, autoclaved corn, and fresh corn groups in the liver and whole blood samples (Figure 2B upper panel). No differences in Ct values were observed between the no-template control group and the three experimental groups in whole blood samples (Figure 2B). Also, after periodate oxidation followed by β elimination, no differences were detected between the no-template control group and the experimental groups in the liver and blood samples (Figure 2B, Table 2).

### 3.3. Analysis of Corn miRNAs in Cecal and Fecal Samples

Corn miRNA was also determined in cecal and fecal samples. In the corn sRNA study, no differences were detected in miRNA Ct values between the control, dNTP, and corn sRNA groups in the cecal or the fecal (from Days 2 and 14) samples. The Ct values of no-template controls for the cecal, fecal (from Days 2 and 14) were significantly higher than those of the experimental groups (Figure 2A). Cecal and fecal (Day 14) samples were then treated with periodate oxidation followed by β elimination. After periodate treatment, no differences were detected between the no-template control group and the experimental groups in cecal samples. However, significant difference persisted in the fecal samples but the differences in Ct values were less (Figure 2A, Table 1). 

A similar observation was made in the cecal and fecal (from Day 2 and 14) samples from the fresh corn powder study. Small but statistically significant differences were detected in miR167a Ct value in cecal and Day 2 fecal samples, and miR164a Ct value in Day 14 fecal samples. No-template control Ct values of cecal and fecal (from Days 2 and 14) samples were significantly higher than those of the experimental groups (Figure 2B). After periodate treatment, differences between the Ct values of the no-template control group and the experimental groups were much smaller compared to those before the periodate treatment in the cecal and the fecal samples (Figure 2B, Table 2). 

Based on the standard curves constructed for each miRNA, the recovery rate of miRNAs from fecal samples were calculated according to the detection levels in qRT-PCR (Table 3 and Table 4). Less than 0.1% of total miRNA (the sum of miR156a, miR164a, and miR167a) tested in both studies were recovered from the fecal samples.

### 3.4. Fate of Corn miRNA in Mouse GI Tract

**Figure 3:**
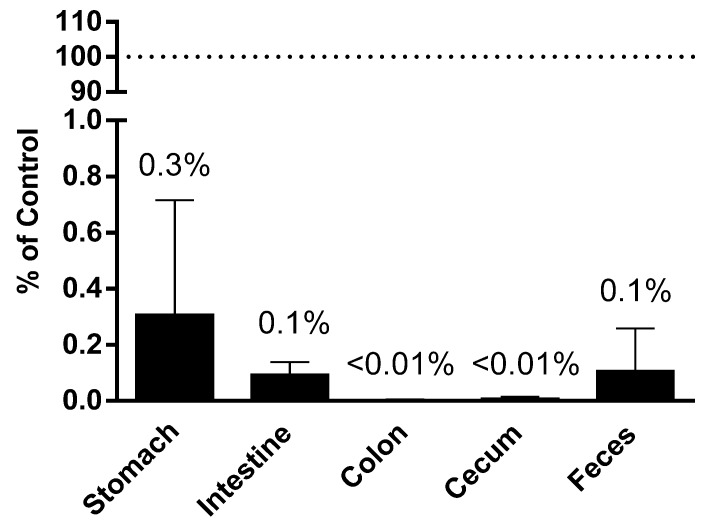
Plant miRNA detection in GI tract and in vitro digestion. Plant miRNA was extracted and quantified from contents of different sections of the GI tract. Recovery was calculated as the percentage of miR167a detected in each section of the GI tract comparing to the total amount detected in the consumed diet.

To assess the effect of digestion on the corn miRNA, corn miRNA was determined in the contents collected from different parts of mouse GI tract from our animal studies. Comparing to the amount of corn miRNA miR167a administered via gavage or food intake, the recovery of miRNAs in each section of the GI tract were calculated to account for less than 0.3% of originally ingested in the stomach, less than 0.1% of originally ingested in the intestine and feces, and less than 0.01% of originally ingested in colon and cecum (Figure 3).

### 3.5. In Vitro Analyses of miRNA Recovery in GI Tract

**Figure 4:**
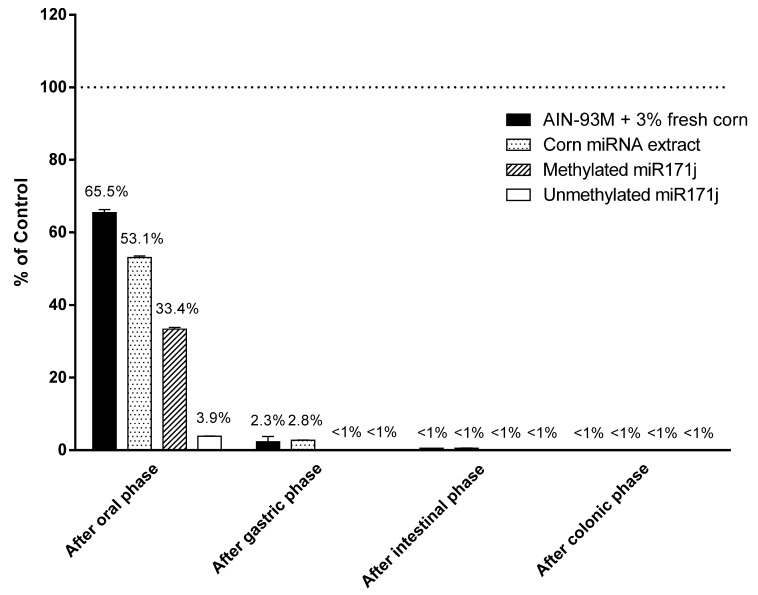
Plant miRNA detection after in vitro digestion. AIN-93M diet supplemented with 3% fresh corn powder, corn miRNA extract, or methylated or unmethylated miR171j were treated with in vitro digestion buffer as described in the Materials and Methods. Recovery was calculated as the percentage of miR167a or miR171j detected after each phase of digestion comparing to the amount measured in the starting materials. All treated samples are significantly different from the respective controls at *p* < 0.05.

Given minimal corn miRNA was accounted for in samples collected, we used an in vitro digestion system to determine if degradation was responsible for the observations. Corn miRNAs supplemented in AIN-93M diet or miRNA extract, as well as methylated and unmethylated synthetic miR171j, were treated following an established in vitro digestion model [25]. After the oral phase, 53–66% of the original corn miR167a were detected in the digestion fluid. Synthetic miR171j with or without 3′ end methylation was used as positive and negative controls and 33% methylated miR171j was recovered, while only 4% of the unmethylated miR171j were recovered after the oral phase (Figure 4). After the gastric phase, over 97% of corn miR167a in AIN diet or in the miRNA extract were degraded. Finally, after the intestinal phase, less than 1% of miR167a was detected in the samples (Figure 4). 

## 4. Discussion

This study investigated the cross-kingdom transfer and bioavailability of plant miRNAs using corn miRNAs administrated in a mouse model and the presence or absence of the ingested miRNAs were analyzed in the diet, cecum, feces, liver, and in whole blood. An updated method using periodate oxidation reaction was employed to ensure the detected miRNA(s) in the biological samples are of plant origin. No detection of corn miRNAs was observed in the cecum, feces, liver, or in the blood following supplementation of corn miRNAs in the animal diet or gavage to the animals. In conjunction with our in vitro digestion study, we concluded that the corn miRNAs are extensively degraded during the digestive process and are not uptake into circulation or tissues in our mouse model.

Recent studies reported conflicting results in the detection of exogenous miRNAs in human or animal models [7,8,9,10,11,12,13,14,15,16,17,18]. Major issues appeared to be (1) the reliability of detection and (2) the biological significance of miRNA at the detected level. The reliability issue of detection may arise from false detection and authenticity of plant origin of the miRNAs. Oversampling in database analyses, potential contamination in sequencing study or false positive detection in PCR assay were pointed out as a possible mechanism of false detection in previous reports [13,14,20]. In this study, we tried to overcome the detection reliability issue by employing a detection method combining the sequence specificity (PCR) and characteristic structure of 3′ end methylation on the plant miRNAs (resistant to periodate oxidation). It is apparent from Figure 2 that potential false positive detections can occur in PCR-based assays, especially at high cycles (>30 cycles). The differences were eliminated by periodate treatments that oxidize unmethylated miRNAs. It is possible that the confounding factor in cecal and fecal samples may be derived from mammalian miRNAs that are similar in sequences or immature plant miRNA that lack the 3′ methylation to protects miRNA from oxidation. A previous study by Luo et al. reported the detection of corn miRNAs in a pig model upon maize and chow consumption [26]. Similar levels of corn miRNAs in the fresh corn were reported by Luo and in this study (Supplementary Materials, Figure S1). Luo and colleagues treated the blood and tissue sample with periodate, which led to reductions in expression levels, while not completely eliminating the detection [26]. The discrepancy between the two studies may stem from the different periodate treatment methods used. Comparing the periodate treatment used in Luo’s [26] studies, which was the same as that used by Zhang and colleagues in the 2012 study [6], periodate oxidation followed by alkaline β elimination used in this study can degrade miRNAs without 3′ end methylation more efficiently [20]. The differences in the model animals and food intake may also contribute to the different results observed in the two studies.

A critical factor for dietary exogenous miRNAs to exert biologically significant effects is the amount of intact and functional miRNAs that actually reaches human or animal circulation and organs. In this study, no detectable levels of corn miRNAs were found in the circulation or the liver (Table 1 and Table 2, Figure 2). In the GI tract, less than 0.1% of corn miRNAs were accounted for and in vitro analysis showed that the dietary miRNAs were quickly and almost completely digested before arriving in the small intestine (Figure 4). Upon losing the intact sequence and the 3′ end methylation, the corn miRNAs may undergo further degradation in the GI tract to individual nucleotides. These nucleotides may be used by gut bacteria or taken up during absorption. However, the amounts of free nucleotides from miRNAs account for a small fraction of the total nucleotides ingested from a dietary source, e.g., genomic DNA and RNA in the food, and is therefore not expected to play an important role as free nucleotides. Therefore, at least for miRNA derived from corn, they are not likely to be available for systemic absorption. Previous studies reported that food matrix, such as exosome packaging of bovine milk miRNAs, can improve the stability of the miRNAs against digestion [27,28]. However, no differences were observed in this study between corn miRNA extract or fresh corn powder supplements. Conflicting evidence of whether certain genetic materials can survive the GI tract was reported in previous studies [29]. Using a different in vitro digestion model consisting of a simulated gastric phase, Philip and colleagues reported that when subjecting soybean seeds to up to 75 min in vitro digestion, soy miRNAs can be detected from the digestion fluid [29]. In this study, a substantial amount of the corn miRNA was degraded in the oral phase, which was missing in Philip’s study. The α-amylase used in this study was prepared from crude human saliva, and therefore, may contain additional enzymes or substances in trace amounts to break down miRNAs. Such reactions may mimic the actual conditions in the oral phase. The extent of degradation in the oral phase may be affected by a number of factors, such as the protective effect of the matrix (AIN-93M diet vs. miRNA extract), the type and amounts of miRNAs (corn miRNAs vs. miR171j), and the methylation status. In other studies, plant miRNAs were observed to be significantly or completely degraded in the GI tract [13,15,17,18]. Considering the absence of detectable corn miRNAs in the circulation and liver in this study and the minimal recovery in the GI tract, the significance and biological relevance of exogenous miRNA transfer may be limited to selected plant foods. 

## 5. Conclusions

Significant degradation of corn miRNAs occurred during digestion which resulted in minimal uptake of corn miRNAs after oral consumption. No corn miRNAs could be detected in whole blood, cecal or liver of the animals. Moreover, degradation of corn miRNAs in the GI tract occurred relatively early and therefore cross-kingdom transfer of exogenous miRNAs appears to be insignificant and not biologically relevant.

## Figures and Tables

**Table 1:** Study 1 miRNA RT-PCR Ct value and calculated concentration after periodate treatment.

**Cecal Sample**												
	**Control**			dNTP **			**Corn miRNA**			**No-Template Control**		
	Cycle #		Calculated conc. (fM) *	Cycle #		Calculated conc. (fM)	Cycle #		Calculated conc. (fM)	Cycle #		Calculated conc. (fM)
miR156a	33.64 ± 0.37	^a^	0.873	33.54 ± 0.40	^a^	0.936	33.75 ± 0.17	^a^	0.809	33.87 ± 0.26	^a^	0.744
miR164a	30.20 ± 0.10	^c^	9.476	30.26 ± 0.13	^bc^	9.090	30.31 ± 0.10	^b^	8.780	30.56 ± 0.07	^a^	7.383
miR167a	32.43 ± 0.21	^a^	2.020	32.51 ± 0.18	^a^	1.911	32.42 ± 0.17	^a^	2.034	32.60 ± 0.09	^a^	1.795
**Day 14 Fecal Sample**												
	**Control**			**dNTP**			**Corn miRNA**			**No-Template Control**		
	Cycle #		Calculated conc. (fM)	Cycle #		Calculated conc. (fM)	Cycle #		Calculated conc. (fM)	Cycle #		Calculated conc. (fM)
miR156a	34.32 ± 0.45	^b^	0.545	34.34 ± 0.41	^b^	0.537	33.94 ± 0.35	^b^	0.709	35.48 ± 0.42	^a^	0.244
miR164a	29.51 ± 0.40	^b^	15.287	29.65 ± 0.25	^b^	13.873	29.52 ± 0.32	^b^	15.181	30.98 ± 0.08	^a^	5.518
miR167a	34.50 ± 0.41	^c^	0.481	34.98 ± 0.39	^ab^	0.345	34.68 ± 0.38	^bc^	0.425	35.24 ± 0.25	^a^	0.288
**Liver**												
	**Control**			**dNTP**			**Corn miRNA**			**No-Template Control**		
	Cycle #		Calculated conc. (fM)	Cycle #		Calculated conc. (fM)	Cycle #		Calculated conc. (fM)	Cycle #		Calculated conc. (fM)
miR156a	35.76 ± 1.31	^a^	0.201	35.32 ± 1.24	^a^	0.272	36.02 ± 1.18	^a^	0.168	36.08 ± 0.46	^a^	0.161
miR164a	34.32 ± 0.40	^a^	0.545	34.58 ± 0.33	^a^	0.455	34.08 ± 0.79	^a^	0.644	35.01 ± 0.30	^a^	0.338
miR167a	34.89 ± 0.49	^a^	0.367	35.03 ± 0.56	^a^	0.333	35.16 ± 0.66	^a^	0.304	35.47 ± 0.51	^a^	0.246
**Whole Blood**												
	**Control**			**dNTP**			**Corn miRNA**			**No-Template Control**		
	Cycle #		Calculated conc. (fM)	Cycle #		Calculated conc. (fM)	Cycle #		Calculated conc. (fM)	Cycle #		Calculated conc. (fM)
miR156a	36.17 ± 1.05	^a^	0.151	36.72 ± 1.13	^a^	0.103	36.50 ± 0.97	^a^	0.120	35.58 ± 1.14	^a^	0.228
miR164a	34.89 ± 0.47	^a^	0.367	34.82 ± 0.43	^a^	0.385	34.90 ± 0.47	^a^	0.365	34.32 ± 0.57	^a^	0.545
miR167a	36.61 ± 0.80	^a^	0.111	36.96 ± 0.87	^a^	0.087	36.93 ± 0.75	^a^	0.089	36.33 ± 0.45	^a^	0.135

* The concentrations were calculated with standard curves of respective microRNAs (miRNAs). Cycle numbers of real-time PCR (RT-PCR) were presented as Mean ± Standard Deviation (*n* = 10). Different letters indicate statistically significant differences (*p* ≤ 0.05); ** Random deoxynucleotide triphosphates.

**Table 2:** Study 2 miRNA RT-PCR Ct value and calculated concentration after periodate treatment.

**Cecal Sample**												
	**Control**			**dNTP**			**Corn miRNA**			**No-Template Control**		
	Cycle #		Calculated conc. (fM) *	Cycle #		Calculated conc. (fM)	Cycle #		Calculated conc. (fM)	Cycle #		Calculated conc. (fM)
miR156a	34.05 ± 0.33	^a^	0.873	33.88 ± 0.26	^a^	0.739	33.95 ± 0.20	^a^	0.704	34.03 ± 0.04	^a^	0.666
miR164a	30.92 ± 0.31	^b^	5.753	31.03 ± 0.08	^ab^	5.330	31.01 ± 0.12	^ab^	5.405	31.15 ± 0.10	^a^	4.905
miR167a	32.41 ± 0.26	^a^	2.048	32.54 ± 0.21	^a^	1.872	32.50 ± 0.19	^a^	1.924	32.37 ± 0.20	^a^	2.106
**Day 14 Fecal Sample**												
	**Control**			**dNTP**			**Corn miRNA**			**No-Template Control**		
	Cycle #		Calculated conc. (fM)	Cycle #		Calculated conc. (fM)	Cycle #		Calculated conc. (fM)	Cycle #		Calculated conc. (fM)
miR156a	33.31 ± 0.41	^b^	1.098	33.40 ± 0.14	^ab^	1.031	33.39 ± 0.27	^ab^	1.038	33.80 ± 0.26	^a^	0.781
miR164a	30.07 ± 0.28	^b^	10.369	30.15 ± 0.18	^b^	9.810	30.15 ± 0.17	^b^	9.810	30.86 ± 0.13	^a^	5.997
miR167a	31.81 ± 0.34	^b^	3.104	31.87 ± 0.19	^b^	2.978	31.93 ± 0.23	^b^	2.856	32.52 ± 0.17	^a^	1.898
**Liver**												
	**Control**			**dNTP**			**Corn miRNA**			**No-Template Control**		
	Cycle #		Calculated conc. (fM)	Cycle #		Calculated conc. (fM)	Cycle #		Calculated conc. (fM)	Cycle #		Calculated conc. (fM)
miR156a	34.55 ± 0.44	^a^	0.465	34.67 ± 0.62	^a^	0.428	34.75 ± 0.28	^a^	0.405	34.76 ± 0.41	^a^	0.402
miR164a	33.42 ± 0.36	^b^	1.017	33.40 ± 0.53	^b^	1.031	33.68 ± 0.33	^ab^	0.849	34.29 ± 0.30	^a^	0.556
miR167a	34.40 ± 0.47	^b^	0.516	34.43 ± 0.47	^b^	0.505	34.62 ± 0.37	^ab^	0.443	35.07 ± 0.39	^a^	0.324
**Whole Blood**												
	**Control**			**dNTP**			**Corn miRNA**			**No-Template Control**		
	Cycle #		Calculated conc. (fM)	Cycle #		Calculated conc. (fM)	Cycle #		Calculated conc. (fM)	Cycle #		Calculated conc. (fM)
miR156a	36.30 ± 0.83	^a^	0.138	36.66 ± 0.78	^a^	0.108	36.47 ± 0.82	^a^	0.123	36.89 ± 1.85	^a^	0.092
miR164a	34.81 ± 0.69	^a^	0.388	34.81 ± 0.63	^a^	0.388	34.90 ± 0.52	^a^	0.365	34.90 ± 0.20	^a^	0.365
miR167a	36.80 ± 0.99	^a^	0.098	36.86 ± 0.46	^a^	0.094	36.69 ± 0.85	^a^	0.105	37.26 ± 1.00	^a^	0.071

* The concentrations were calculated with standard curves of respective miRNAs. Cycle numbers of RT-PCR were presented as Mean ± SD (*n* = 10). Different letters indicate statistically significant differences (*p* ≤ 0.05).

**Table 3:** miRNAs recovered in fecal sample from study 1.

miRNA	Intake from Gavage	Detected in Fecal Sample	% Recovered
miR156a	56.03 pg	0.27 pg	0.48%
miR164a	13.2 pg	0.25 pg	1.89%
miR167a	1215.63 pg	0.39 pg	0.03%

**Table 4:** miRNAs recovered in fecal sample from study 2.

miRNA	Intake from Diet	Detected in Fecal Sample	% Recovered
miR156a	575.56 pg	0.31 pg	0.05%
miR164a	217.36 pg	1.00 pg	0.46%
miR167a	1716.44 pg	0.73 pg	0.04%

## Supplementary Materials

The following are available online at http://www.mdpi.com/2072-6643/10/2/215/s1, Figure S1: Stability of corn miRNAs in the AIN-93M diet at the start and end of the feeding period, Figure S2: Degradation of corn miRNAs after autoclaving, Table S1: Diet composition.
