# Supplementary material for: Extensive Degradation and Low Bioavailability of Orally Consumed Corn miRNAs in Mice
Source: Nutrients. 2018 Feb 15;10(2):215. doi: 10.3390/nu10020215 (PMC5852791; doi:10.3390/nu10020215)
Supplement: Supplementary file 1 [file nutrients-10-00215-s001.pdf]

SUPPLEMENTAL INFORMATION:

Supplemental Table 1. Diet composition

**Diet group**	**AIN93M base diet**	**AIN93M+3% fresh corn powder**	**AIN93M+3% autoclaved corn powder**
**Ingredient**	**gm**	**gm**	**gm**
Casein	140	135.9	135.9
L-Cystine	1.8	1.8	1.8
Corn Starch	495.692	473.8	473.8
Maltodextrin 10	125	125	125
Sucrose	100	97	97
Cellulose, BW200	50	47.5	47.5
Soybean Oil	40	39.1	39.1
t-Butylhydroquinone	0.008	0.008	0.008
Mineral Mix S10022M	35	35	35
Vitamin Mix V10037	10	10	10
Choline Bitartrate	2.5	2.5	2.5
**Fresh corn powder**	0	**30**	0
**Autoclaved corn powder**	0	0	**30**
**Total**	**1000**	**997.608**	**997.608**
**Fresh corn powder %**	**0.00**	**3.01**	**0.00**
**Autoclaved corn powder %**	**0.00**	**0.00**	**3.01**
**gm**			
Protein	125.0	125.0	125.0
Fat	40.0	40.0	40.0
Toal Carbohydrate	730.7	730.7	730.7
Sugar	100.0	100.0	100.0
Fiber	50.0	50.0	50.0
**kcal**			
Protein	500	500	500
Fat	360	360	360
Total Carbohydrate	2923	2923	2923
Sugar	400	400	400
Total kcal	**3783**	**3783**	**3783**
**kcal%**			
Protein	13	13	13
Fat	10	10	10
Total Carbohydrate	77	77	77
Sugar	11	11	11
Total	100	100	100
**kcal/gm**	**3.78**	**3.79**	**3.79**

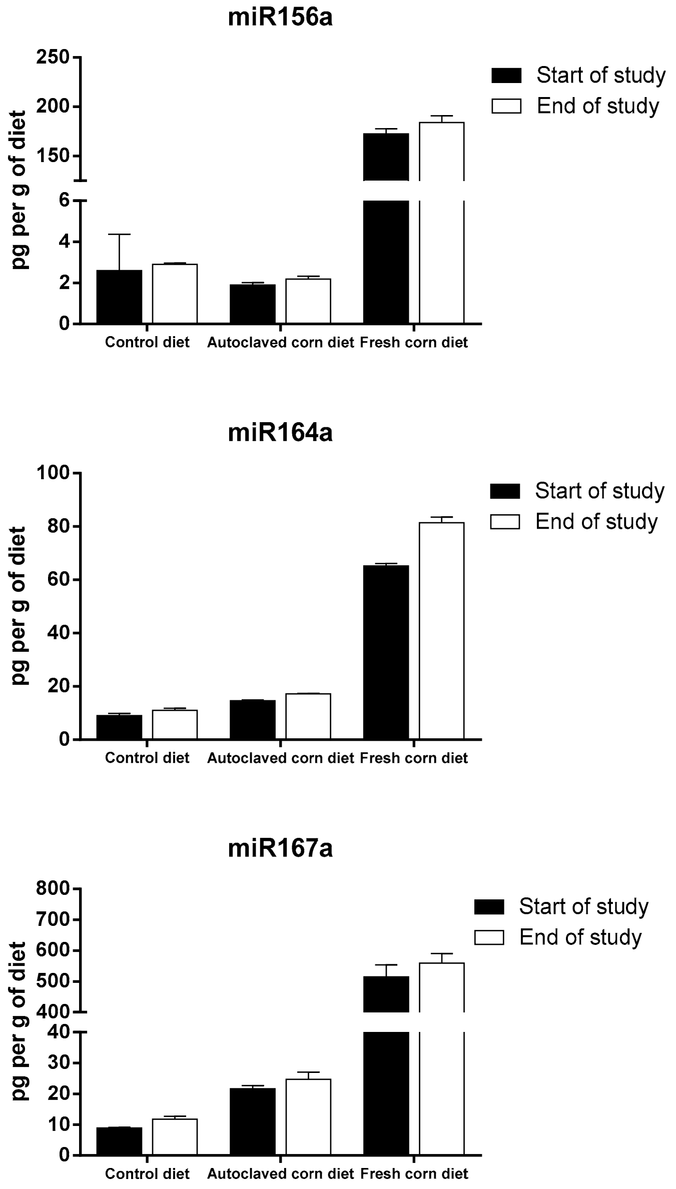

Supplemental Fig. 1 Stability of corn miRNAs in the AIN-93M diet at the start and end of the feeding period. MiRNAs were extracted from AIN-93M diets at the start or end of the feeding period. The relative concentrations of miR156a, miR164a, and miR167a were determined using RT-PCR.

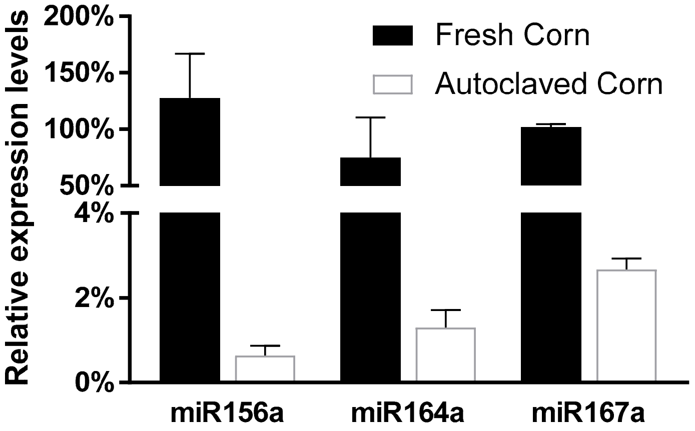

Supplemental Fig. 2 Degradation of corn miRNAs after autoclaving. Fresh corn kernels were autoclaved at 121 °C for 30 min. Then miRNAs were extracted from fresh corn kernels or autoclaved corn kernels. The relative concentrations of miR156a, miR164a, and miR167a were determined using RT-PCR.
